# Genetic evolution of parental populations and construction of core germplasm populations in qinghai spruce seed orchard based on SLAF-seq technology

**DOI:** 10.3389/fgene.2025.1712106

**Published:** 2025-11-17

**Authors:** Hu Zhao, Erwen Xu, Dong Lv, Wei Li, Xingpeng Zhao, Xin Jia, Hao Yuan, Rong Zhou

**Affiliations:** 1 Academy of Water Resource Conservation Forests of Qilian Mountains in Gansu Province, Zhangye, China; 2 College of Biology and Biotechnology, Beijing Forestry University, Beijing, China

**Keywords:** qinghai spruce, seed orchards, SLAF-seq technology, parental populations, genetic evolution, core germplasm

## Abstract

In order to explore the genetic background and genetic basis of the parental population of Qinghai spruce (*Picea crassifolia* Kom.) seed orchard, to reduce the cycle of genetic improvement of Qinghai spruce and the scale of germplasm resources, and to enhance the level of genetic improvement of Qinghai spruce. This study utilized the SLAF-seq technology to conduct genetic and evolutionary analysis on 165 germplasms from 11 provenances of Qinghai spruce seed orchards, and developed a total of 1964178 high-consistency single nucleotide polymorphism markers. Phylogenetic analysis classified them into three major groups, while population structure analysis revealed two subgroups. Through the Core Hunter II software, 33 (20%) core germplasms were selected, retaining all genetic diversity. This research provides a scientific basis for the genetic improvement of Qinghai spruce.

## 1 Introduction

The application of genetic improvement in conifers is primarily focused on seed orchards. Seed orchards serve as a foundation for the propagation of superior forest tree seeds or as a direct source of substantial quantities of high-quality seeds for production and afforestation. They represent a pivotal element in the ongoing enhancement of the breeding system, progressing from the lower level to the higher level. It is evident that forest trees exhibit varying degrees of heterozygosity. Consequently, the enhancement of forest trees’ genetic composition must be conducted in a progressive and cyclical manner, utilising breeding activities such as selection, genetic testing and configuration design. Each population type should be employed as the material for these activities, thereby facilitating multi-generational genetic enhancement (Wu et al., 2023). The first seed gardens were established in China during the 1960s; however, they experienced a period of stagnation in the 1970s, and many have not been well preserved or maintained. In the late 1970s, China initiated the construction of primary seed gardens on a large scale, with the establishment of more than 10 important coniferous species of primary seed gardens (Zhang et al., 2013), including the Qinghai spruce asexual seed orchards in Zhangye City, Gansu Province, which was established in 1984. Since then, the research unit of the Academy of Water Resource Conservation Forests of Qilian Mountains in Gansu Province has conducted extensive research on the asexual seed orchards of Qinghai spruce. For instance, the construction of seed orchards, the determination of half-sibling progeny, and the selection of optimal family lines (Gu., 2001), as well as the asexual seed orchard flowering habit (Lei et al., 2012), the asexual seed orchard pollen dispersal and spatial distribution (Li et al., 2012), and the response of phenology of asexual lineages to meteorological factors (Kang et al., 2013), asexual male and female bulbous flowers and bulbous fruit volume variation (Lv et al., 2013), asexual flowering characteristics and spatial and temporal variations of seed orchard pollen flow (Lv et al., 2016), and studies on the genetic variation of fruiting traits in asexual seed orchard lines (Zhao et al., 2017). However, these efforts have been lingering in the 1st generation seed orchard stage, and there is an urgent need to accelerate the improved generation of Qinghai spruce using modern forest breeding strategies.

The advent of molecular marker technology has led to its widespread utilisation in population genetics research. This field involves the analysis of a substantial quantity of precise nucleotide variation information across the entire genome, employing advanced mathematical and statistical methodologies. The objective is to facilitate discourse on the genetic underpinnings of population genetics. This approach facilitates the analysis of the genetic structure of populations, the mechanisms underlying species formation, the dynamics of gene exchange, and the evolutionary processes of populations. It also provides a theoretical foundation for breeding practices, thus contributing to the advancement of plant evolutionary research (Wang, 2007; Zhou et al., 2011). In the domain of population genetics studies of forest trees, molecular marker techniques commonly employed include simple sequence repeats (Duan et al., 2020; Zhong et al., 2018; Jurkšienė et al., 2019), amplified fragment length polymorphic markers (Di et al.,. 2020), and random amplified polymorphic DNA (RAPD). Conventional methods of marker development yield a limited number of markers, lack representativeness, and necessitate substantial manpower and time. Simplified genome sequencing represents an approach for large-scale molecular marker development based on high-throughput sequencing platforms. In comparison with conventional marker development methodologies, this approach offers several advantages, including high throughput, automation, and high accuracy. Furthermore, it facilitates the simultaneous acquisition of molecular markers and genotyping data, thus rendering it the most prevalent method in population genetics research (Lu et al., 2019). Site-specific amplified fragment sequencing (SLAF-seq) technology is a simplified genome sequencing technique that has been extensively utilised in recent years in population genetics studies of uninvolved species. SLAF-seq technology facilitates the development of genome-wide molecular markers in a short period of time and at a low cost, and it is currently one of the most popular techniques for molecular marker development. The technique is characterised by high throughput, long effective reads, high accuracy, good reproducibility and low cost (Sun et al., 2013). Chen Lijie et al. utilised SLAF-seq technology to obtain 283,376 highly consistent population single nucleotide polymorphism (SNP) markers from 48 Guizhou ancient tea tree (Camellia sinensis) resources. The population structure analysis based on these SNPs was able to clearly classify the ancient tea tree resources into two taxa, arboreal and shrubby (Chen et al., 2019). Duan Yizhong et al. employed SLAF-seq technology to obtain 102025 highly consistent SNPs of sardonyx populations, and carry out genetic analysis of Ammopitanthus mongolicus in 10 locations (Duan et al.,. 2018); Yao Lei used SLAF-seq technology to carry out certain analyses on the genetic diversity and genetic evolution of China fir (Cunninghamia lanceolata (Lamb.) Hook.), which provided a certain scientific basis for the classification, collection, preservation and evaluation of cedar germplasm resources as well as the development and selection of cedar germplasm resources (Yao, 2017). It is evident that SLAF-seq has become a reliable tool in population genetics studies of uninvolved species.

Qinghai spruce is a primary afforestation species in the Qilian Mountains of northwestern China. It plays a vital ecological role in soil and water conservation, while also serving as a key pillar of the local forestry economy. In recent years, the ecological environment of Qilian Mountain in the northwest of China has undergone significant improvement, with the Qinghai spruce becoming a primary afforestation and greening tree species. This has led to an increased demand for the refinement of quality and quantity in the production of its fine breeds. Consequently, there is an urgent need to upgrade Qinghai spruce fine breeds and to undertake fundamental research on the selection of high-generation parents of Qinghai spruce and genetic evaluation. A primary challenge is the extensive collection of Qinghai spruce germplasm resources, which is currently preserved using a comprehensive approach. This method is characterised by its lack of focus and pertinence, resulting in difficulties in the preservation and efficient utilisation of germplasm resources. Secondly, while there has been some research conducted on the genetic variation of Qinghai spruce germplasm resources and genetic improvement, only limited progress has been made in the selection and breeding of superior varieties. Traditional cross-breeding and rotational selection are still the primary methods employed, and the required improvement cycle is too extensive, making it impractical to select and breed genetically exceptional varieties in a brief period.

In order to reduce the cycle of genetic improvement of Qinghai spruce and the scale of germplasm resources, and to enhance the level of genetic improvement of Qinghai spruce, this study takes 165 germplasm from 11 provenances parental populations of Qinghai spruce asexual seed orchard as the material, adopts the SLAF-seq technology, obtains a large number of polymorphic SLAF tags, then develops a number of high consistency of the SNP loci, and carries out the genetic diversity analysis of Qinghai spruce, to explore its The genetic evolution history and survival status of Qinghai spruce was then combined with SNP molecular marker data to compare the evaluation parameter values of stepwise clustering priority sampling method and minimum distance stepwise clustering method based on different genetic distances and genetic similarity coefficients, and to explore the optimal strategy for constructing a molecular core germplasm population of Qinghai spruce, so as to provide a scientific method for the preservation of Qinghai spruce germplasm resources and the selection and breeding of the target traits, and at the same time to provide a way of thinking for the construction of core germplasm populations of other coniferous tree resources. At the same time, it also provides an idea for other coniferous resources to construct core germplasm populations.

## 2 Materials and methods

### 2.1 Test materials

**FIGURE 1:**
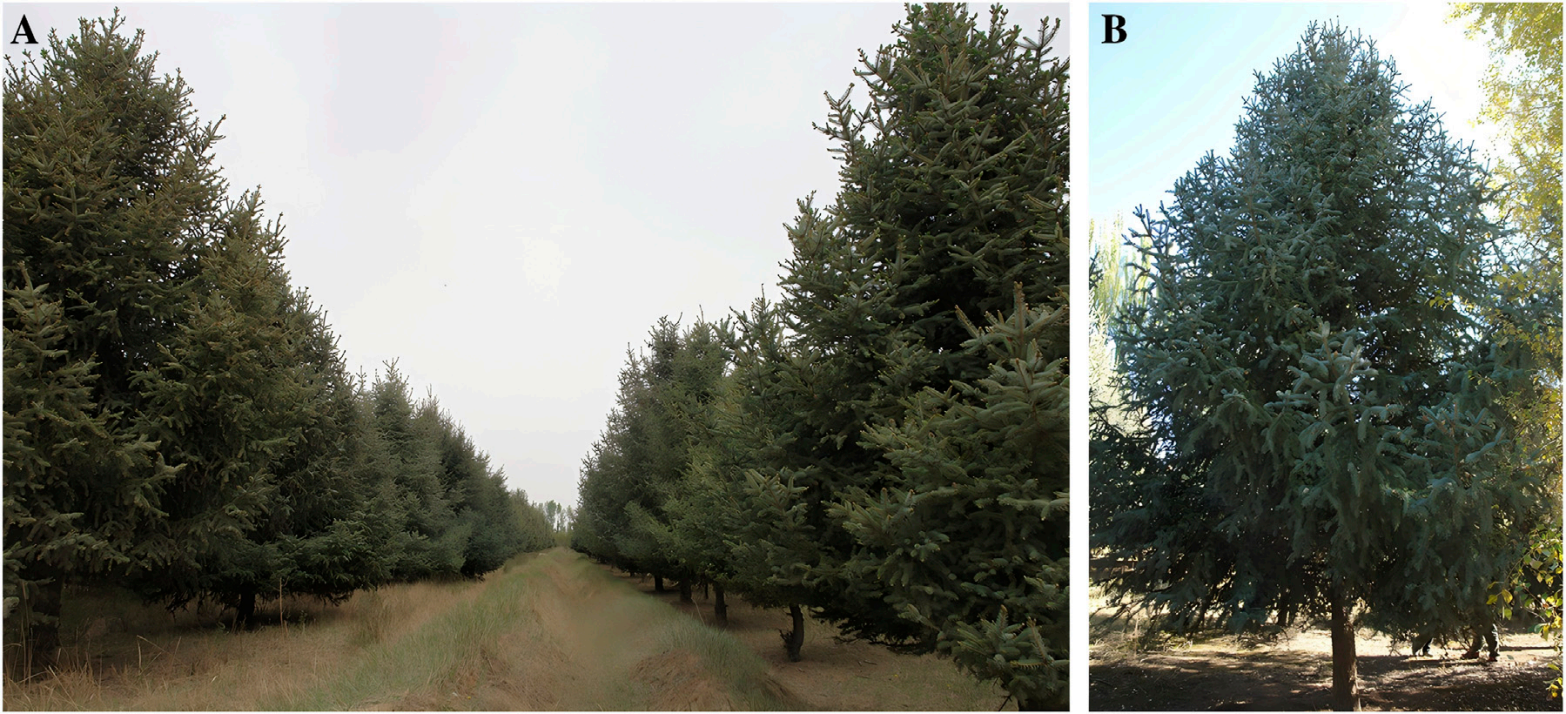
Qinghai spruce asexual seed Orchard. **(A)** A corner of Qinghai spruce asexual seed Orchard; **(B)** Excellent single plants of Qinghai spruce.

**FIGURE 2:**
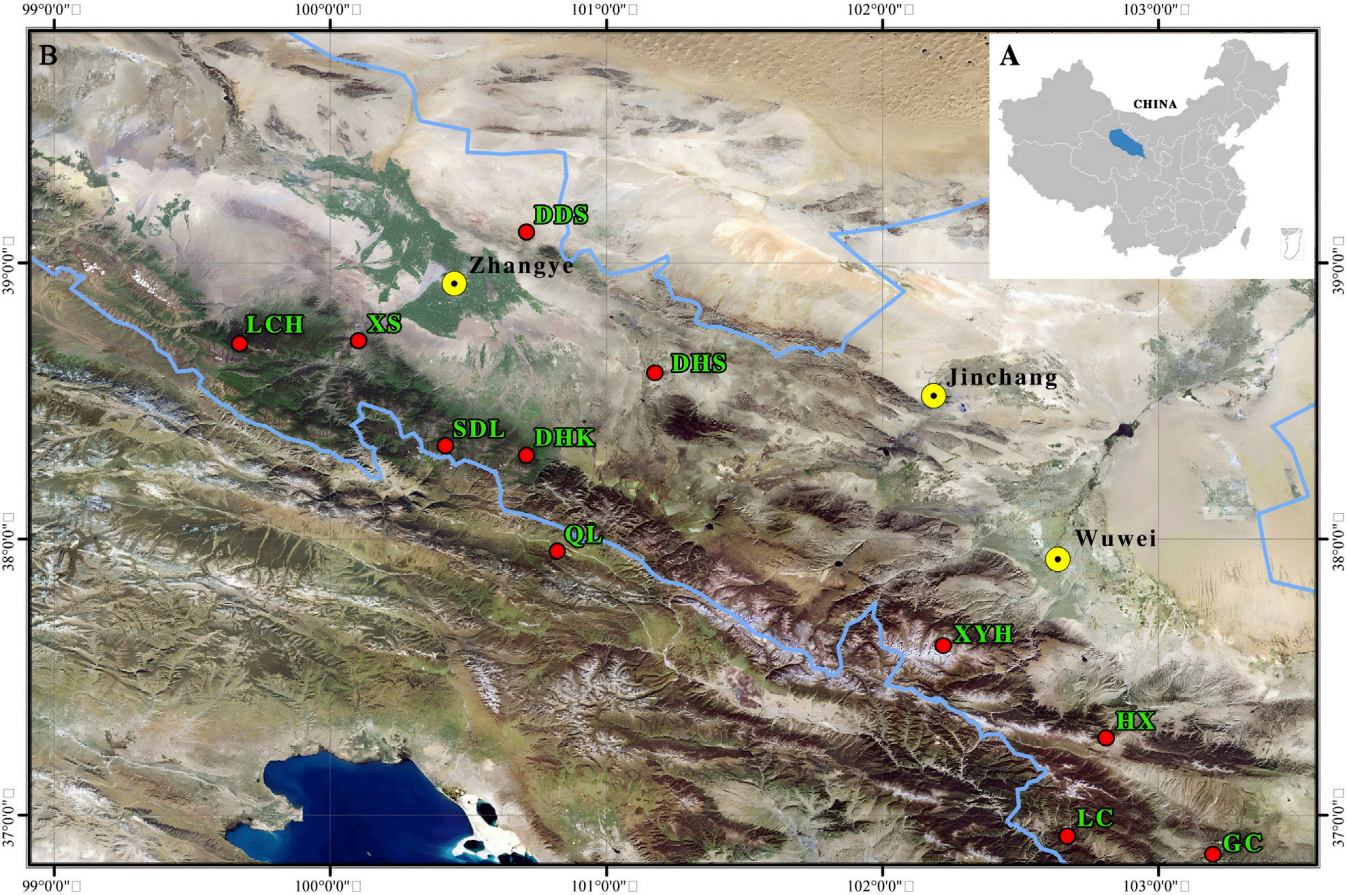
Qinghai spruce provenance distribution map. **(A)** Location of Qilian Mountains in China; **(B)** Location of 11 Qinghai spruce provenances from the Qilian Mountains.

The experimental materials were obtained from the Qinghai spruce asexual seed orchard of the National Key Forest Tree Seed Base in Zhangye City, Gansu Province, China (Figure 1), and the materials for the establishment of the Qinghai spruce asexual seed orchard were obtained from 11 forest farms in the Qilian Mountain region, namely, Xishui, Liancheng, Daihuangshan, Haxi, Dongdashan, Longchanghe, Gucheng, Dahakou, Qilian, Xiyinghe and Sidalong (Figure 2). A total of 165 excellent asexual lines of Qinghai spruce were collected and planted in a completely randomised field design by grafting on existing green spruce rootstocks at the base. The pith layer affixation method was grafted onto the existing Qinghai spruce rootstocks at the base, and a completely randomised field design was adopted for planting, with each asexual line being planted with nine plants and repeated three times. In the year 2023, 165 parental populations from 11 provenances of the Qinghai spruce asexual seed orchard (40a) were selected for the study. Healthy needles from the current year of each parental single plant were collected in freezing tubes, promptly stored in liquid nitrogen, brought back indoors, and placed in the refrigerator for future use. The material information is outlined in Supplementary Table S1.

### 2.2 Genomic DNA extraction and testing

Genomic DNA was extracted using a modified CTAB method, with the specific steps comprising: grinding 0.5 g of needle leaves in liquid nitrogen, adding pre-warmed CTAB extraction buffer at 65 °C, incubating in a 65 °C water bath for 1 hour, chloroform-isopentanol extraction, isopropanol precipitation, washing with 70% ethanol, drying, and dissolving in 50 μL of TE buffer. The concentration of the extracted Qinghai spruce genomic DNA was not less than 20 ng/L, and the total amount was not less than 4 g, to ensure that the quality was in accordance with the requirements for the establishment of the library.

### 2.3 SLAF library construction and high-throughput sequencing

In the absence of sequence information for the Qinghai spruce genome, the Picea abies genome was selected as the reference genome (http://plantgenie.org/Data/ConGenIE/Picea_abies/v1.0/). The Picea abies genome was assembled into a 12G file with a GC content of 37.88%. Subsequent to the assembly of the Picea abies reference genome, an e-enzymatic digestion prediction was performed to determine the optimal digestion scheme (Davey et al., 2012).

The genomic DNA of each sample with adequate quality was enzymatically digested, and digest the genomic DNA with HinCII. After performing the 3′-end adenylation treatment, ligate the Dual-index sequencing adapters (Kozich et al., 2013). Then, construct the library through PCR amplification. The reaction system for PCR is 25 μL, which consists of 2.5 μL of 10× PCR buffer, 2 μL of dNTP, 1 μL of each primer, 50 ng of DNA template, 0.5 μL of KOD-Plus-Neo, and the volume is made up to 25 μL with ddH_2_O. The target fragments were selected through the steps of purification, mixing, and gel cutting, and then subjected to bipartite sequencing with Illumina HiSeq after the library was qualified by the quality inspection. Oryza sativa ssp. japonica was selected as the control for the library construction, and the genomic data were obtained from http://rapdb.dna.affrc.go.jp. The construction and sequencing of the SLAF library was conducted by Beijing Baimike Biotechnology Co.

### 2.4 SLAF sequencing data analysis and SNP site screening

The Raw Reads thus obtained were then subject to quality control, with the objective of obtaining Clean Reads. The obtained reads were then compared to the reference genome using BWA software (Li and Durbin et al., 2009) in order to count the number of SLAF tags and polymorphic SLAF tags. The development of SNP molecular markers was achieved through the utilisation of both GATK (McKenna et al., 2010) and Samtools (Li et al., 2009) software, with minor genotype frequency (MAF) > 0.05 and completeness >0.8 serving as the screening criteria. The intersection of the two software-developed SNP markers was then employed as the final reliable SNP marker.

### 2.5 Analysis of genetic diversity

Genetic diversity was analysed for each locus, with the following parameters being calculated: minor allele frequency (MAF), expected number of alleles (Ne), expected heterozygosity (He), Nei diversity index, observed heterozygosity (Ho), polymorphism information content (PIC) and Shannon diversity index (I). The indices of each point of the samples within the population were then averaged to calculate the population value. The value of the inbreeding coefficient (Fis) was calculated for each sample using the Vcftools-het command, and the samples within the population were averaged as the population value. The coefficient of genetic differentiation (Fst) was calculated using the PopGenome software package.

### 2.6 Analysis of genetic evolution

The construction of the phylogenetic tree for each sample was achieved by means of the MEGA X (Kumar et al., 2018) software, with the neighbour-joining method (neighbour-joining) being utilised as the basis for this process. The p-distance distance calculation model was employed to calculate the distances between the samples. The analysis of the population structure of the study materials was conducted utilising the admixture (v1.22) software (Alexander et al., 2009), based on single-nucleotide polymorphisms (SNPs). For the study population, the number of subgroups (K-value) was predetermined to be 1-10 for the purpose of clustering. The results of the clustering process were subjected to cross-validation, and the optimal number of clusters was determined based on the valley of the cross-validation error rate. The genetic structure of the Qinghai spruce population was investigated by utilising admixture software. The number of subpopulations (K value) was pre-set as ranging from 1 to 10, and the clustering process was performed. Subsequently, the cross-validation error rate on the clustering results was calculated in order to determine the optimal number of subpopulations based on its valley value. The samples were clustered using the smartpca programme in the EIGENSOFT (v6.0) (Price et al., 2006) package, which is based on SNP data and performs principal component analysis. The utilisation of the GCTA (v1.92.1) (Yang et al., 2011) software facilitates the estimation of kinship between two individuals within a natural population. The linkage disequilibrium between two two-single-nucleotide polymorphisms (SNPs) within a specified distance range (10 kilobases) on the same chromosome was calculated using PopLDdecay (v3.41) (Zhang et al., 2019) software. The strength of linkage disequilibrium was expressed as r2. The closer the r2 is to 1, the stronger the strength of chain disequilibrium is represented. Each population genetics index was calculated according to the specified window and step size using the VCFtools (v0.1.15) (Danecek et al., 2011) software, based on the high-consistency SNPs.

### 2.7 Analysis of genetic evolution

Core Hunter II (Thackuk et al., 2009) is capable of extracting a diverse, representative subset with minimal redundancy from a large number of germplasm resources to construct core germplasm or microcore germplasm (Li et al.,. 2019). Based on the SNP data, weighted with a variety of assessment measures (Modified Rogers distance, Shannons Diversity Index, etc.), the screened material has been shown to exhibit high diversity, high representativeness and high allelic richness.

In order to conduct this analysis, Core Hunter II software was utilised in conjunction with the weighted indices Modified Rogers distance (0.7) and Shannons Diversity Index (0.3), in accordance with the total germplasm proportion 0.1, 0.2. , 0.3, 0.4, 0.5, 0.6, 0.7, 0.8, 0.9 gradient. Screening was then carried out, after which the screened materials were evaluated for gene coverage (CV).

## 3 Results

### 3.1 Library sequencing results

The *Picea_abies*:v1.0 genome was selected as the reference genome for e-enzymatic prediction, and it was finally determined that HinCII enzyme digestion was used, and sequences with enzyme section lengths in the range of 314–394 were defined as SLAF tags. A total of 3856.98 Mb reads were obtained. 3802489 SLAF tags were obtained by bioinformatics analysis, of which a total of 668397 polymorphic SLAF tags were obtained, and a total of 12271817 population SNPs were obtained. The sequencing reads of the rice Nihon Haru genome after RasI + Hin CII digestion were compared with its reference genome by Soap software, and the efficiency of double-end comparison was 94.78%. The percentage of residual enzyme cleavage sites in the inserted fragments of the statistical rice Nihon Haru sequencing reads was 89.93%, indicating a high efficiency of enzyme cleavage. The results of both double-end matching efficiency and enzymatic efficiency evaluation of rice Nihonkari data indicated that this SLAF library construction was normal.

A total of 3856.98 Mb reads were obtained in this experiment. The base distribution all showed that the first 2 bases of the reads showed base separation consistent with the enzyme cut site, and the subsequent base distribution would show different degrees of small fluctuations, composite DNA enzyme section of the base distribution characteristics. The number of reads Q30 and GC content of the sequencing data were counted, and the number of reads of each sample ranged from 5000384 to 61110406. The GC content ranged from 39.19% to 43.61%, with an average GC content of 40.23%, and the Q30 ranged from 86.91% to 99.96%, with an average Q30 of 96.34%.

### 3.2 SLAF markers and SNP information statistics

Bioinformatics analysis yielded a total of 3802489 SLAF tags, of which 668397 (17.58%) were found to be polymorphic. The number of SLAF tags contained in each sample ranged from 150536 to 708176, and the average sequencing depth of the SLAF tags in the samples was 19.74x.

A total of 12271817 population SNPs were obtained, and the number of SNPs detected in each sample ranged from 2806119 to 6862965, with SNP heterozygosity ranging from 13.41% to 20.52% and SNP completeness ranging from 22.87% to 55.92%.

The application of filters for minor allele frequency (MAF: 0.05) and locus completeness (INT: 0.5) yielded a total of 1964178 highly concordant population single-nucleotide polymorphisms (SNPs) for subsequent genetic evolutionary correlation analyses.

### 3.3 Analysis of population genetic diversity

As demonstrated in Supplementary Table S2, the genetic diversity analysis revealed that the minor allele frequency (MAF) of all SNP loci in the 165 germplasm resources ranged from 0.21 to 0.35, with a mean value of 0.25. The expected number of alleles (Ne) ranged from 1.379 to 1.480, with a mean value of 1.447; the expected heterozygosity (He) ranged from 0.298-0.426 with values less than 0.5, indicating that all populations were subjected to high-intensity selection; Nei diversity index (Ndi) ranged from 0.302-0.568 with a mean value of 0.369; number of polymorphic markers (Npm) ranged from 516,257-194,681,515; The number of observed alleles (No) ranged from 1.496-1.991, with a mean value of 1.817; observed heterozygosity (Ho) ranged from 0.230-0.601, with a mean value of 0.338, indicating that the number of heterozygotes accounted for a relatively small proportion of the population; polymorphism information content (PIC) ranged from 0.243-0.334, with a mean value of 0.270; And Shannon diversity index (I) ranged from 0.460-0.616, with a mean value of 0.506. As demonstrated by the analysis of the genetic diversity index, the 165 Qinghai spruce germplasm resources exhibited a collective degree of genetic diversity. The variation in genetic diversity among the 11 populations was found to be non-significant, a phenomenon that may be associated with artificial selection and the planting environment.

### 3.4 Analysis of population genetic evolutionary

This analysis was based on the SNP data from variant detection, filtered according to minor allele frequency (MAF:0.05) and locus completeness (INT:0.5), and high concordance SNP loci (Num:1964178) were obtained for phylogenetic analysis, population structural analysis, and population principal component analysis. These analyses were used to reveal the genetic differentiation relationship of Qinghai spruce from multiple aspects.

#### 3.4.1 Phylogenetic analysis

**FIGURE 3:**
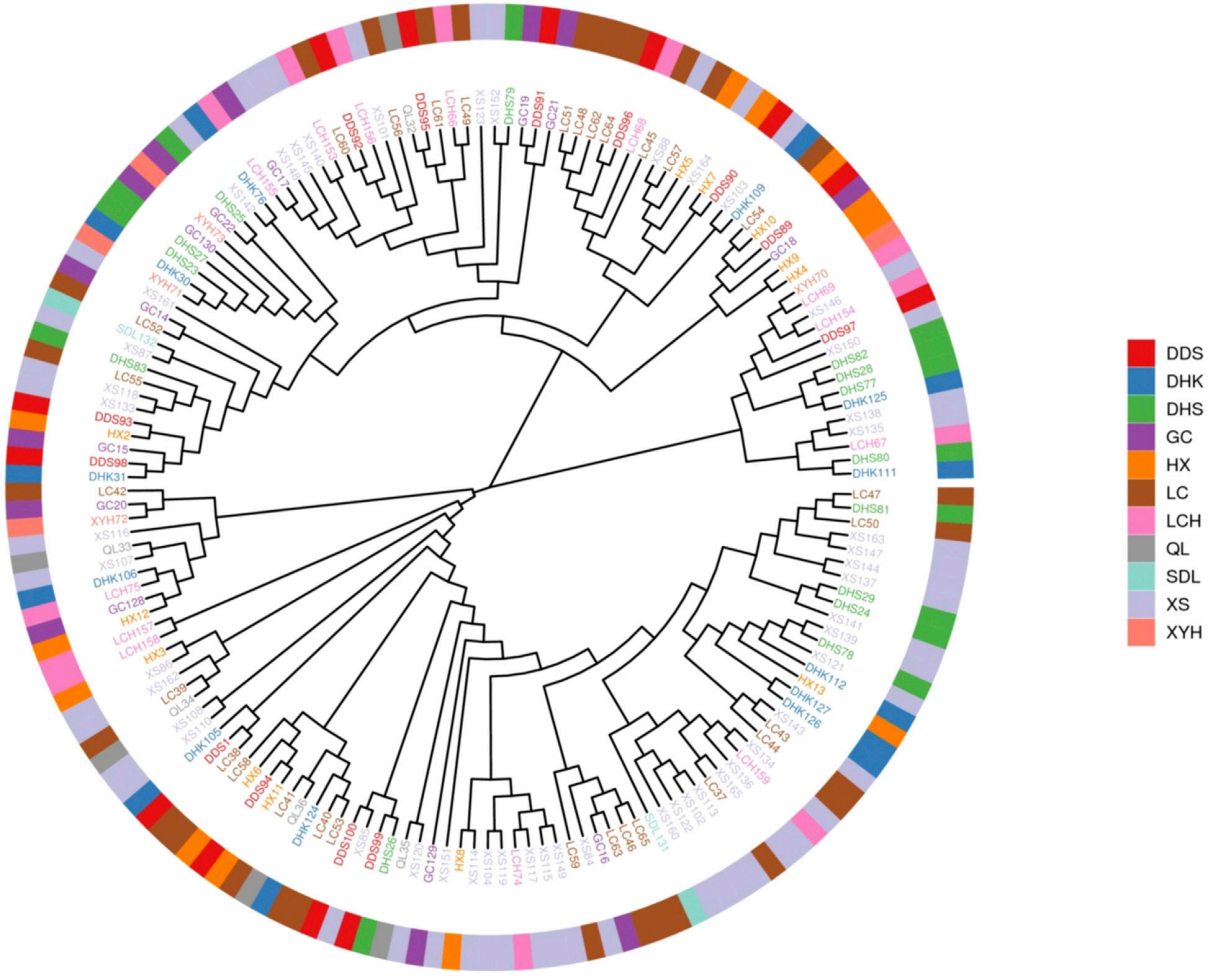
Phylogenetic tree of 165 germplasm materials of Qinghai spruce.

A phylogenetic tree of Qinghai spruce was constructed using the developed high-quality Qinghai spruce SNP markers (Figure 3). The study classified 165 germplasms into three major groups, which contained 15, 66 and 84 Qinghai spruce monocots, respectively. The first major group (P1) comprised six seed sources from Xiyinghe (XYH), Longchanghe (LCH), Xishui (XS), Dongdashan (DDS), Daihuangshan (DHS), and Dahekou (DHK) (Ndi-0.348). The second major group (P2) consisted of 10 seed sources from Xiyinghe (XYH), Longchanghe (LCH), Xishui (XS), Dongdashan (DDS), Daihuangshan (DHS), Dahekou (DHK), Haxi (HX), Gucheng (GC), Liancheng (LC), and Sidalong (SDL) (Ndi-0.364), and the third major group (P3) consisted of Xiyinghe (XYH). The third major group (P3) consisted of 11 provenances (Ndi-0.369) from Xiyinghe (XYH), Longchanghe (LCH), Xishui (XS), Dongdashan (DDS), Dahuangshan (DHS), Dahuankou (DHK), Haxi (HX), Gucheng (GC), Liancheng (LC), Sidailong (SDL), and Qilian (QL). This finding suggests that the phylogenetic grouping was not entirely congruent with the geographic origin of the germplasm.

#### 3.4.2 Analysis population genetic structure and principal component

**FIGURE 4:**
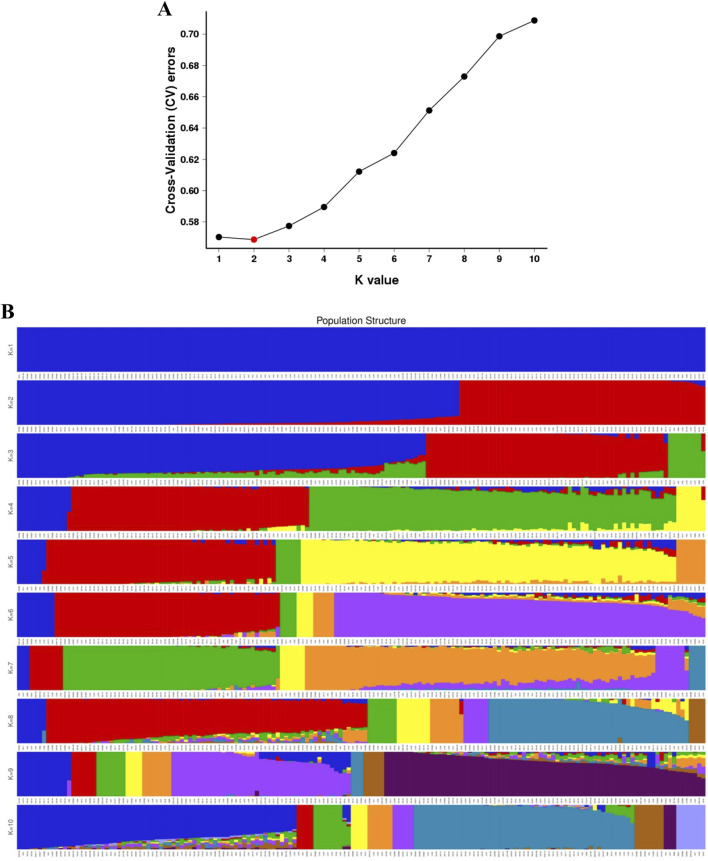
**(A)** Cross-validation error rates for various K values; **(B)** Individual clustering plots for various K values.

The population structure of 165 Qinghai spruce asexual lines was analysed by using Admixture (v1.22) software based on the SNP markers. The results showed that the cross-validation error rate was the lowest when the value of K = 2 (Figure 4A), the optimal number of subgroups was 2, which indicated that the possibility of the 165 monocultures originating from two ancestors was relatively high, and that they could be divided into two subpopulations, with 60 samples being divided into a subgroup (G1) and 105 samples into one subpopulation (G2) (Figure 4B).

**FIGURE 5:**
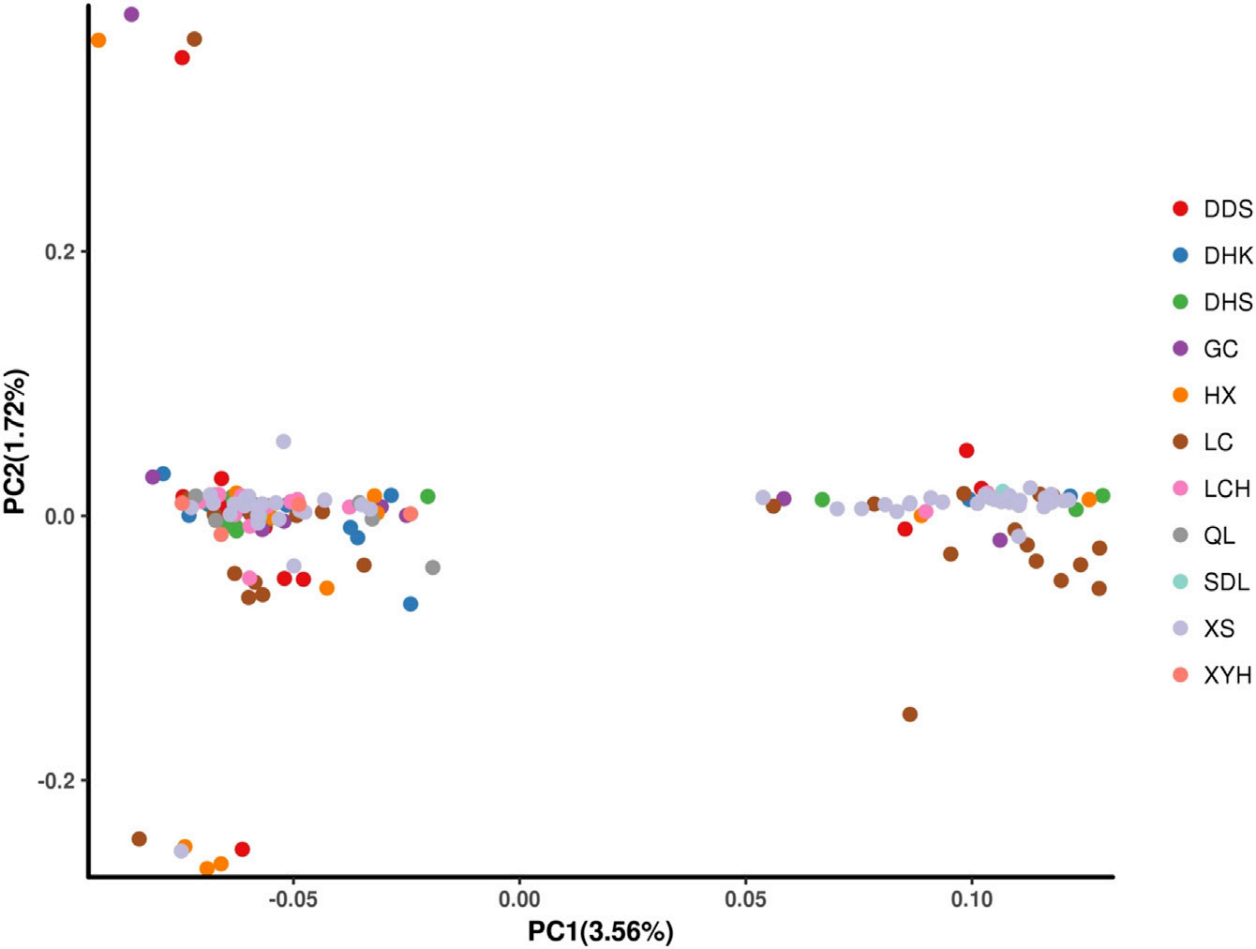
Principal component analysis (PCA).

The principal component analysis demonstrated that the 165 samples were predominantly distributed across two distinct clusters, with only a limited number of individual samples exhibiting deviation from these two groups (Figure 5). This finding is in alignment with the outcomes of the population genetic structure analysis. This finding is consistent with the results of the population genetic structure analysis, and each taxon contains asexual lines of different geographical origins, which further corroborates the results of the phylogenetic and population structure analyses.

### 3.5 Core germplasm construction and evaluation

#### 3.5.1 Core germplasm construction

**FIGURE 6:**
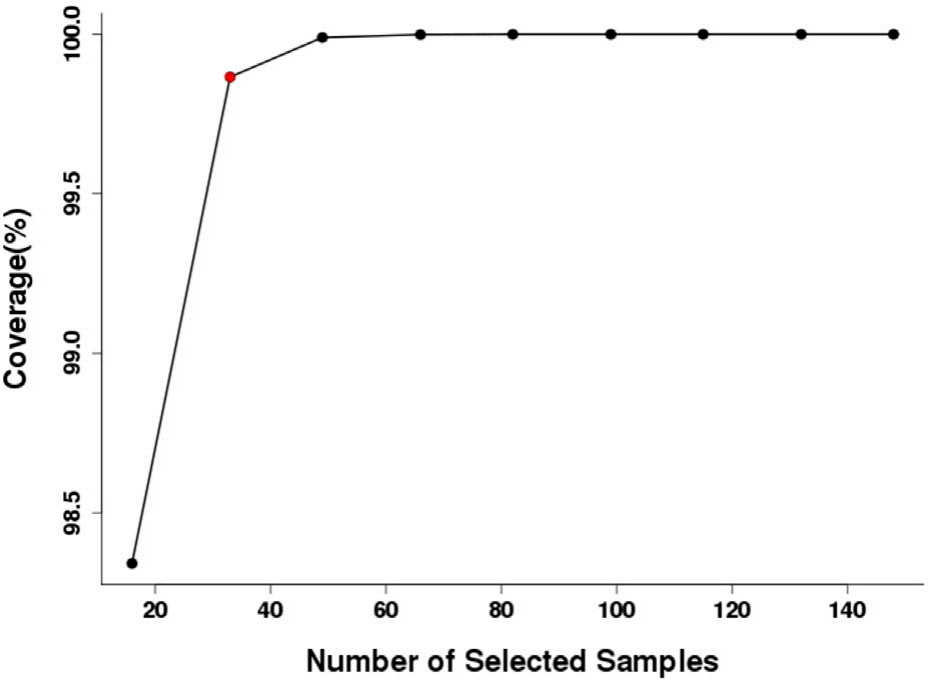
Gene coverage evaluation of the core collection of Qinghai spruce.

Core Hunter II software was utilised to further calculate the genetic diversity of the parental population in the Qinghai spruce seed orchard with a view to capturing the core germplasm. The analysis demonstrated that CORE 0.5 was the core germplasm population that maximised the retention of genetic variation in the entire breeding population with a minimum number of asexual lines, including a total of 33 asexual lines (Supplementary Table S3). The ratio of the number of core germplasm individuals to the total number of individuals in the original population was 20%, and gene coverage was evaluated for the screened material (Figure 6), with the result that gene coverage was greater than 99.8%.

#### 3.5.2 Core germplasm assessment

##### 3.5.2.1 Genetic diversity assessment

The results presented in Supplementary Table S4 demonstrate that the number of core germplasm samples accounted for 20% of the original germplasm samples, yet retained the entirety of the genetic diversity of the original germplasm. The mean values of observed heterozygosity, expected heterozygosity, Nei diversity index, Shannon-Wiener index, and polymorphic information content for the original germplasm were 0.255, 0.262, 0.263, 0.419, and 0.219, respectively; for the core germplasm, they were 0.291, 0.284, 0.289, 0.448, and 0.236, respectively; and for the core germplasm, these five genetic parameters were retained as 114.12%, 108.40%, 109.89%, 106.92% and 107.76%, respectively. The mean values of the five genetic diversity evaluation parameters of the core germplasm in this study were higher than those of the original germplasm, indicating that the constructed core germplasm eliminated the genetic redundancy of the original germplasm and maximally preserved the genetic diversity of the original germplasm, and therefore met the requirements of the core germplasm.

##### 3.5.2.2 Allele assessment

**FIGURE 7:**
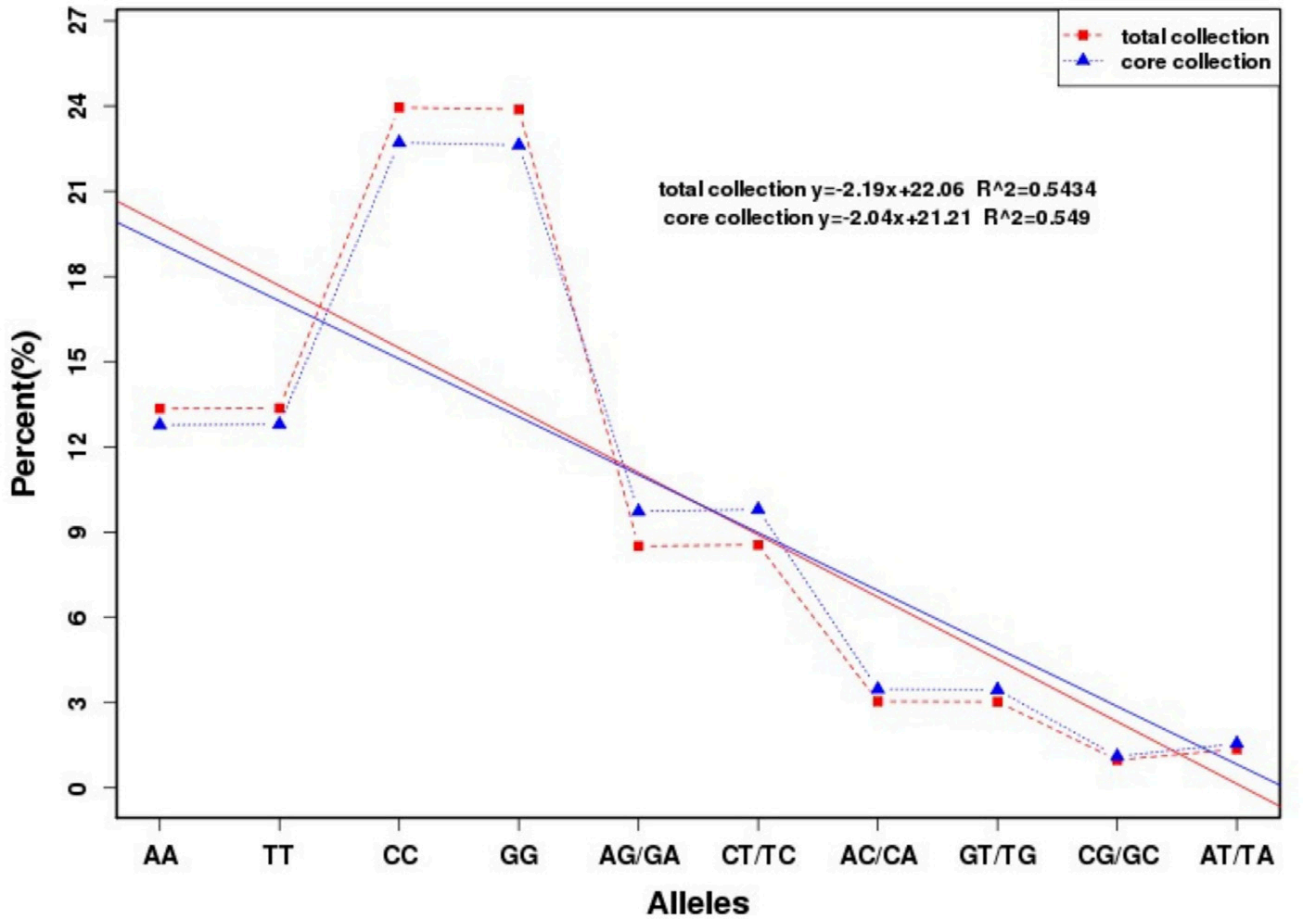
Distribution of the genotype ratio of the original and core germplasm of the Qinghai spruce.

The wide range as well as complexity of germplasm resources determines that there is no universal sampling ratio, and different species with different research needs also determines that there is no perfect ratio and fixed size for all core germplasm sets. In the current study, if almost all alleles appear in the core germplasm set, it also predicts that the screened core germplasm meets the optimal sampling ratio. In this experiment, four alleles, A, C, G, and T, produced four pure and six heterozygous, for a total of 10 genotypes. The distribution of the number of the 10 allele types produced in the original and core germplasm is shown in Supplementary Table S5, and the distribution of the proportions is shown in Figure 7. From Figure 7, it can be seen that the core germplasm retained almost 100% of the frequencies of the 10 alleles of the original germplasm, in which the retention proportion of the pure genotypes CC and GG was 98%, AA and TT was 99%, and the retention proportion of the rest of the genotypes was greater than or equal to 100%, and it can be seen that the genotype distribution curves of the original and the core germplasm and the fitting curves of the original spruce and the core germplasm basically coincided with each other.

**FIGURE 8:**
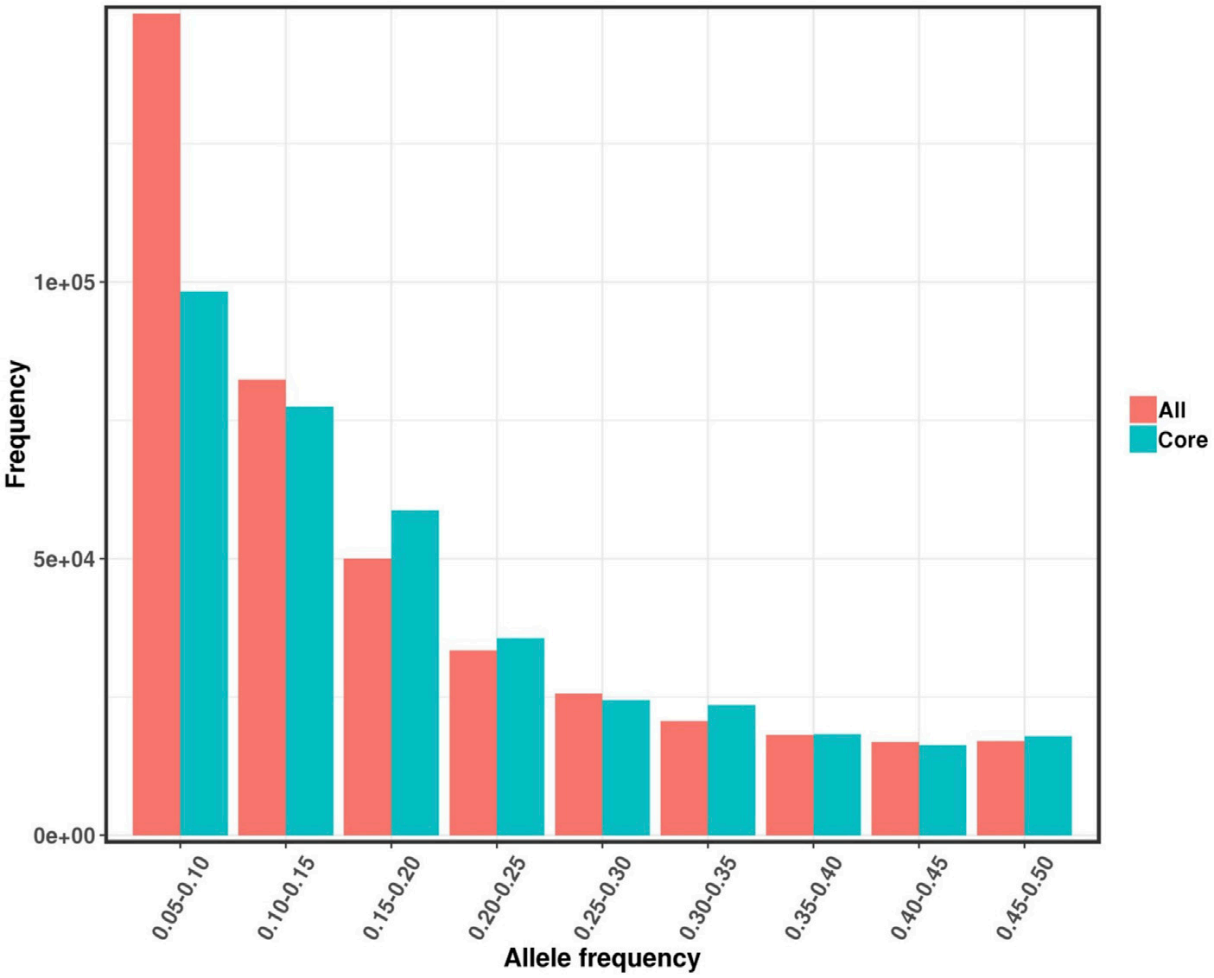
Distribution of minor alleles in the original and core germplasm of Qinghai spruce.

The distribution of minor alleles in the original and core germplasm of Qinghai spruce is shown in Figure 8. The distribution of minor alleles between 0.05-0.10 in the original germplasm was significantly larger than that in the core germplasm, while the distribution of the remaining MAFs in the original and core germplasm differed very little, which also reflected the reasonableness of the screening of the core germplasm as well as the accuracy from the side.

##### 3.5.2.3 Principal component analysis (PCA)

**FIGURE 9:**
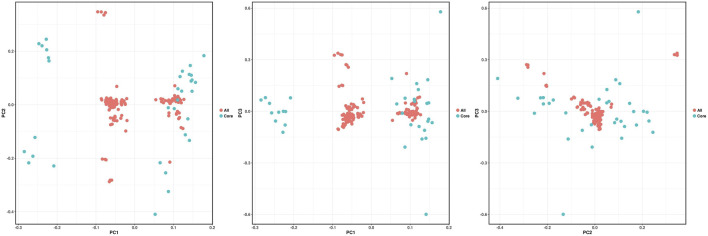
Cluster diagram of original and core germplasm of Qinghai spruce by principal component analysis.

The accuracy of the core germplasm screening was assessed by principal component analysis of the original Qinghai spruce germplasm materials and the screened core germplasm materials. In principle, the reasonableness of the screening results is indicated by the fact that the trends of the principal component plots drawn based on the core germplasm and the distribution plots of all materials coincide. From the three two-dimensional principal component cluster plots (Figure 9), it can be seen that the trends of the distribution of core germplasm and original germplasm basically coincide, indicating that the screened core germplasm is well represented.

## 4 Discussion

### 4.1 Genetic relationships research of parental populations based on SNPs

Simplified genome sequencing is very suitable for some species without reference genomes, which can well solve the problems of genome complexity and lack of sequence marker information, thus enabling the development and use of large-scale SNP markers (Danecek et al., 2011). SNP molecular markers have the characteristics of wide distribution, high polymorphism, high heritability, etc., which are widely used in high-throughput screening, and have been widely applied in genetic diversity analysis, genetic evolution, kinship, population structure analysis, QTL localisation, and construction of genetic linkage maps, etc. (Rafalski, 2002; Mammadov et al., 2012; Jehan and Lakhanpaul, 2006). Pengle Li determined 293 samples of Pinus massoniana using simplified genome sequencing (GBS) technology, carried out a genome-wide selection GS breeding study for Pinus massoniana GBS-SNP development, and established a GS breeding technology system for Pinus massoniana based on GBS sequencing SNP development (Li, 2022). Mingliang Dong used simplified genome sequencing (SLAF-seq) technology for large-scale SNP marker development and constructed a high-density genetic linkage map of Larix principis-rupprechtii Mayr, and carried out QTL analyses for growth and needle traits in Larix principis-rupprechtii Mayr, obtaining some QTLs controlling important phenotypic traits (Dong, 2020). Yali Wang obtained 105685557 high-quality SNPs by whole genome resequencing of Xanthoceras sorbifolium Bunge, and performed population genetics analysis of Xanthoceras sorbifolium Bunge (Wang and Li et al., 2022). Dongya Qiao et al. performed simplified genome sequencing of 85 Lagerstroemia indica germplasm resources using SNP markers, obtained SNP typing data, and confirmed that the 85 Lagerstroemia indica germplasm resources were rich in genetic diversity, and there was high gene flow among different taxa (Qiao et al., 2020). The number of SNP markers developed in this study (1964178) is significantly higher than that reported by Yuan Xingdong (Yuan, 2016) using SSR markers (14.76 effective alleles), but lower than that reported by Hu Yang (Hu, 2016) using ISSR markers (Shannon-Wiener index of 0.3502). This discrepancy may be related to marker types and germplasm scale.

In this study, we carried out simplified genome sequencing of 165 Qinghai spruce germplasm resources with reference to the *Picea abies* genome, and analysed the genetic relationship and genetic structure of the parental groups of Qinghai spruce seed gardens at the genome level. The results of phylogenetic analysis, population structure analysis and principal component analysis were basically consistent and complementary to each other, and the parental groups of the 165 Qinghai spruce seed gardens were divided into three major taxa; the results of phylogenetic analysis, population structure analysis and grouping were not completely consistent with the geographic origin of these parental groups. The results of phylogenetic analysis, population structure analysis and principal component analysis were basically consistent and complementary to each other, and the 165 Qinghai spruce seed orchards were divided into three major taxa; the phylogenetic analysis, population structure analysis and the geographic origins of these parental populations were not completely consistent, and in general, the germplasm of the same geographic origin was both relatively aggregated, but also mixed in distribution, and was not completely grouped; Suggests that populations from the same geographic provenances are both related to each other and to germplasm resources from other taxa. This is mainly because the population used in this experiment is mainly obtained from the Qilian Mountains screening of good single plants, provenances, etc. In the process of long-term selection, the germplasm of different provenances also exchanged resources, and this germplasm exchange led to the results of the genetic analysis of the population and the geographical origin of the correlation is not significant. Similar findings were reported by Yanyan Wang et al. that there was no significant correlation between the grouping results of 113 cigar tobacco grass population structure analyses and the geographic origin of germplasm resources (Wang et al., 2021). Similarly, a study on *Gossypium hirsutum* L. by Qiujin Tan et al. showed that *G. hirsutum* L. germplasm of different origins were intertwined in the same cluster and were not differentiated according to geographic origin (Ai et al., 2017).

In this study, although the results of phylogenetic analysis, population structure analysis, and principal component analysis are generally consistent, certain discrepancies still exist. For example, the phylogenetic tree classifies the population into three major groups, whereas the population structure analysis reaches its optimal state when K = 2, and more mosaic patterns within subgroups emerge when K = 3. This inconsistency may indicate the presence of frequent gene flow and bidirectional introgression of Qinghai spruce in the Qilian Mountains region. The sampling sites cover a complex mountainous terrain that extends approximately 550 km from east to west and 250 km from north to south, encompassing multiple ridges and river valleys. Geographical isolation and topographical variations may not have completely hindered the dissemination of pollen and seeds. Pollen clouds dispersed by wind and seed spread along valley watercourses may have promoted gene exchange among different subgroups, leading to a genetic structure featuring an intertwined pattern of “interpenetration”. Despite the absence of specific meteorological data, the prevailing wind direction, seasonal variations in wind strength, and the flow directions of rivers in the Qilian Mountains region are likely to have a significant influence on the direction of gene flow. In future research, integrating geographic information systems and climate data is expected to more accurately unveil the role of the environment in shaping the genetic structure.

### 4.2 Genetic diversity research of parental populations based on SNPs

For a species, genetic diversity is the basis for its survival, development and evolution, determines its evolutionary potential and adaptability to the environment, and responds to its potential to be modified and utilised (Govindaraj et al., 2015), and is an important component of plant genetics, breeding, conservation and evolution (Qiao et al., 2020). Currently, studies on genetic diversity based on phenotypic traits, SSR molecular markers, and ISSR molecular markers have been carried out in Qinghai spruce (Wang, 2007; Wang, 2009; Wu et al., 2024; Yuan, 2016; Huang et al., 2022), whereas studies using SNP markers to analyse the genetic diversity of Qinghai spruce have rarely been reported.

In this study, we used 165 Qinghai spruce whole genome SNP data and analysed the genetic diversity of 165 subgroups of Qinghai spruce population structure. For the two subgroups of population structure, the mean values of suballele frequency, expected allele number, expected heterozygosity, Nei diversity index, observed allele number, observed heterozygosity, and Shannon-Wiener index were 0.25, 1.447, 0.334, 0.369, 1.817, 0.338, 0.506, respectively; where the genetic diversity index of the was sorted as subgroup G1 > G2. It indicates that the genetic diversity of the 165 Qinghai spruce seed orchard parental populations used in this study is at a medium-high level. Xingdong Yuan analysed the genetic diversity of 109 Qinghai spruce asexual populations using SSR molecular markers, and the effective allele number 14.7601, expected heterozygosity 0.9925, and Shannon-Wiener index 2.7747 were higher than those investigated using SNPs in the present study; this is mainly due to the fact that SNPs have double alleles, and the expected heterozygosity and so forth are usually lower reason (Yuan, 2016). Yang Hu analysed the genetic diversity of 125 Qinghai spruce asexual populations using ISSR technique, and the effective alleles, expected heterozygosity and Shannon-Wiener index were 1.2843, 0.2074 and 0.3502, respectively, which were very similar to the resultant values in this study (Hu, 2016).

### 4.3 Construction and evaluation of core germplasm populations

Preserving a large number of germplasm resources can provide a rich genetic basis for genetic improvement and variety selection of Qinghai spruce, but Qinghai spruce is a tall conifer with high tree height and large land area, which brings certain difficulties to the preservation of Qinghai spruce germplasm resources. Constructing a core germplasm population of Qinghai spruce can preserve more germplasm resources on limited land and achieve the goal of genetic diversity preservation (Huang et al., 2022). At present, the construction of core germplasm is mainly based on the means of phenotypic traits or molecular markers, or the use of phenotypic traits combined with molecular markers (Parida et al., 2014). Mingkun Chen et al. used SSR molecular markers to construct 51 *Cymbidium ensifolium Germplasm* core germplasm, which accounted for 16.4% of the original germplasm resources and could represent the genetic diversity of *C. ensifolium Germplasm* resources to the greatest extent (Chen et al., 2022). Hongguo Li et al. used SSR molecular markers based on the allele number maximisation strategy to obtain 189 core germplasm of *Eucommia ulmoides*, with a sampling proportion of 21.3%, and the core germplasm retained 100% of the alleles and genotypes of the original germplasm (Li, 2017).

The construction of core germplasm of Qinghai spruce has not been reported. In this study, based on 165 simplified genome sequencing SNP data from the parental population of Qinghai spruce seed orchards, using Core Hunter II software, combined with the weighted index Modified Rogers distance (0.7) and Shannons Diversity Index (0.3), 33 samples (20% of the original germplasm) were selected as core germplasm according to the stepwise compression method, which retained all the genetic diversity of the original germplasm. The effectiveness of Core Hunter II software for constructing core germplasm has been validated and applied to fast-growing, high-quality fir and grape crops (Duan et al., 2017).

An effective and well-represented core germplasm can maximise the retention of genetic diversity of the original germplasm. The wide range as well as complexity of germplasm resources determines that there is no universal sampling proportion, and Brown et al. recommended a sampling proportion of 20%–30% as core germplasm for germplasm resources (Brown and Schoen, 1994). Zichao Li et al. posited that factors such as population size and genetic structure of different species affect the sampling proportion of core germplasm (Li et al.,. 2003). Zheng et al. advanced the argument that for different species, different research needs, as well as the degree of collection of original germplasm, genetic diversity, etc., determine that there is no perfect proportion or fixed size of core germplasm (Zheng et al., 2019). In the context of empirical research, the proportion of core germplasm to original germplasm in various crops is predominantly between 10%–30% (Wang J. et al., 2014; Miao et al., 2016). Consequently, in the process of constructing core germplasm, researchers customarily adjust the sampling proportion according to the crop species, as well as the size and scale of the original population. However, Roland Schafleitner et al. have highlighted the importance of directly selecting the backbone parents or varieties (lines) that have played a significant role in production and possess outstanding characteristics, as well as special germplasm with exceptional traits, into the core germplasm bank, in order to prevent the loss of valuable germplasm or genes (Schafleitner et al., 2015). The core germplasm screened in this study accounted for 20% of the original germplasm, which retained all the genetic diversity of the parental population in the Qinghai spruce seed orchard and could represent the parental population better. However, given the limitation of the 165 germplasm materials employed in this study, which precludes their capacity to fully represent the totality of Qinghai spruce germplasm resources, the core germplasm constructed in subsequent studies must be subject to appropriate adjustment and updating as new resources are collected. This process of refinement will involve the incorporation of novel germplasm resources as they become available.

Whether the constructed core germplasm is effective or not needs to be evaluated with the help of a series of core germplasm genetic diversity evaluation parameters. The following are some of the most commonly used parameters for the evaluation of the genetic diversity of core germplasm: allele number, minor allele frequency, observed heterozygosity, expected heterozygosity, Nei diversity index, Shannon-Wiener index, polymorphic information content, etc. Hongguo Li et al. considered that allele number is the most relevant indicator for detecting the validity of core germplasm, and that maximising allele number implies preserving the most genetically diverse germplasm resources (Li, 2017). Jiancheng Wang et al. considered Shannon-Wiener diversity index, polymorphic information content and Simpson’s index as important parameters for evaluating whether the core germplasm is representative or not (Wang Y. et al., 2014). Heterozygosity is the probability that two randomly selected samples in a population have alleles that are not identical, and a representative core germplasm should have a heterozygosity similar to that of the original germplasm (Agrama et al., 2009). In this study, the observed heterozygosity, expected heterozygosity, Nei diversity index, Shannon-Wiener index, polymorphic information content, allele and minor allele frequencies were utilised to evaluate the core germplasm. The genetic coverage of the constructed core germplasm was greater than 99.8%, and almost 100% of the alleles and genotypes of the original germplasm were retained. The retention ratios of genetic parameters such as observed heterozygosity, expected heterozygosity, Nei diversity index, Shannon-Wiener index, and polymorphic information content were 114.12%, 108.40%, 109.89%, 106.92%, and 107.76%, respectively. These ratios were utilised to maximise the preservation of the genetic diversity of the original germplasm, thereby verifying the validity of the constructed core germplasm. The validation of the constructed core germplasm is satisfactory. Representative core germplasm has been shown to maximise the retention of genetic diversity of the original germplasm. This feature can be reflected not only in the values of multiple genetic parameters, but also in scatter plots of material distribution based on principal component analysis. There have been several reports on comparative studies of distributional characteristics of core germplasm and original germplasm using principal component analysis (Xu et al., 2008; Boczkowska et al., 2016; Nayak et al., 2014). In order to perform a more comprehensive evaluation of the effectiveness of the core germplasm construction of the parental population of the Qinghai spruce seed orchard, this study also carried out a principal component assessment based on the evaluation of the genetic diversity parameters. The results confirmed that the principal component maps drawn based on the core germplasm and the principal component maps of the original germplasm exhibited a distribution trend coinciding with that of the core germplasm maps. This further illustrated the reasonableness and effectiveness of the screening results.

The core germplasm constructed in this study retained all the genetic diversity of the original germplasm with only 20% of the sample proportion, indicating that there is a high degree of genetic redundancy in the parent population of the Qinghai spruce seed orchard. This redundancy may result from frequent gene flow and hybridization, leading to high genetic similarity among individuals from different geographical origins. Although the sampling points covered multiple forest farms, long-term artificial selection, grafting propagation, and gene exchange during natural pollination may have weakened the influence of geographical distance on genetic differentiation. Therefore, in the construction of the core germplasm, the genetic distance based on SNP is more representative than geographical origin. Moreover, the core germplasm still retains representative individuals from all 11 provenances, indicating that the constructed core germplasm has good coverage in both genetic and geographical dimensions.

## 5 Conclusion

The results of the present study’s phylogenetic, population structure and principal component analyses were found to be largely consistent and complementary. The 165 Qinghai spruce seed orchard parental populations were divided into three major taxa (Q1, Q2 and Q3). However, there was a lack of complete consistency between the taxa groupings and the groupings of the geographic origin of the germplasm; the 165 Qinghai spruce seed orchard parental populations used exhibited a medium-high level of genetic diversity, and the population structure analyses divided the experimental population into two subgroups (G1 and G2), with G1 exhibiting higher genetic diversity than G2.

Utilising the SNP data of 165 Qinghai spruce seed Orchards, a core germplasm of Qinghai spruce comprising 33 samples (screening ratio of 20%) was constructed. The gene coverage of the core germplasm was found to exceed 99.80%, thereby ensuring the retention of nearly 100% of the alleles and genotypes of the original germplasm, while also preserving the entirety of the genetic diversity of the original germplasm. The distribution trend of the principal component map based on the core germplasm was found to be in alignment with that of the original germplasm. The distribution trend of the principal component map based on the core germplasm and the original germplasm coincided, indicating that the constructed core germplasm exhibited adequate representativeness, thus providing a foundation for the gene conservation of Qinghai spruce germplasm resources. It is recommended that future research endeavours focus on broadening the scope of germplasm collection and incorporating genome-wide association studies (GWAS) to identify the genes that regulate significant traits. This approach will facilitate the development of precise molecular marker-assisted selection tools, which will in turn enable the genetic enhancement of Qinghai spruce.

## Data Availability

The original contributions presented in the study are publicly available. This data can be found in NCBI with the accession number PRJNA771805 here: https://www.ncbi.nlm.nih.gov/bioproject/PRJNA771805.
