# Supplementary material for: Genetic evolution of parental populations and construction of core germplasm populations in qinghai spruce seed orchard based on SLAF-seq technology
Source: Front Genet. 2025 Nov 17;16:1712106. doi: 10.3389/fgene.2025.1712106 (PMC12665348; doi:10.3389/fgene.2025.1712106)
Supplement: Supplementary file 1 [file Table1.doc]

Supplementary Material

# Supplementary Tables

**Table 1.** Experimental material information

| **Material sources** | **Quantity** | **Longitude** | **Latitude** | **Elevation(m)** |
| --- | --- | --- | --- | --- |
| XS | 49 | 100°03′~100°23′ | 38°23′~38°48′ | 2735 |
| LC | 29 | 102°26′~102°55′ | 36°33′~36°48′ | 2790 |
| DHS | 14 | 101°00′~101°30′ | 38°20′~38°30′ | 2752 |
| HX | 12 | 102°01′~102°51′ | 37°16′~37°45′ | 2613 |
| DDS | 13 | 100°45′~100°51′ | 39°00′~39°04′ | 2712 |
| LCH | 13 | 99°32′~100°01′ | 38°39′~38°56′ | 2589 |
| GC | 12 | 102°26′~102°51′ | 36°45′~37°07′ | 2595 |
| DHK | 12 | 100°31′~100°56′ | 37°54′~38°31′ | 2468 |
| QL | 5 | 102°06′~102°25′ | 37°31′~37°51′ | 2485 |
| XYH | 4 | 102°24′~102°66′ | 36°57′~37°25′ | 2605 |
| SDL | 2 | 99°53′~100°07′ | 38°26′~38°48′ | 2830 |

***** Xishui forest area (XS); Liancheng forest area(LC); Daihuangshan forest area(DHS); Haxi forest area(HX); Dongdashan forest area(DDS); Longchanghe forest area(LCH); Gucheng forest area(GC); Dahekou forest area(DHK); Qilian forest area(QL); Xiyinghe forest area(XYH); Sidalong forest area(SDL)

**Table 2. Genetic diversity index of different populations of Qinghai spruce**

| **Populations** | **MAF** | **Ne** | **He** | **Ndi** | **Npm** | **No** | **Ho** | **PIC** | **I** |
| --- | --- | --- | --- | --- | --- | --- | --- | --- | --- |
| DDS | 0.23 | 1.458 | 0.317 | 0.336 | 1,715,797 | 1.874 | 0.290 | 0.258 | 0.485 |
| DHK | 0.23 | 1.454 | 0.320 | 0.340 | 1,682,584 | 1.857 | 0.289 | 0.260 | 0.489 |
| DHS | 0.23 | 1.464 | 0.315 | 0.332 | 1,747,026 | 1.889 | 0.286 | 0.256 | 0.483 |
| GC | 0.23 | 1.457 | 0.319 | 0.338 | 1,696,193 | 1.864 | 0.300 | 0.259 | 0.488 |
| HX | 0.23 | 1.462 | 0.319 | 0.338 | 1,713,502 | 1.872 | 0.306 | 0.259 | 0.487 |
| LC | 0.22 | 1.480 | 0.302 | 0.310 | 1,905,505 | 1.970 | 0.248 | 0.247 | 0.466 |
| LCH | 0.23 | 1.459 | 0.318 | 0.335 | 1,714,318 | 1.873 | 0.300 | 0.258 | 0.485 |
| QL | 0.28 | 1.415 | 0.366 | 0.419 | 1,260,983 | 1.664 | 0.411 | 0.292 | 0.545 |
| SDL | 0.35 | 1.379 | 0.426 | 0.568 | 516,257 | 1.496 | 0.601 | 0.334 | 0.616 |
| XS | 0.21 | 1.480 | 0.298 | 0.302 | 1,946,815 | 1.991 | 0.230 | 0.243 | 0.460 |
| XYH | 0.29 | 1.412 | 0.377 | 0.444 | 1,178,807 | 1.636 | 0.455 | 0.300 | 0.559 |

***** Dongdashan forest area(**DDS**), Dahekou forest area(**DHK**), Daihuangshan forest area(**DHS**), Gucheng forest area(**GC**), Haxi forest area(**HX**), Liancheng forest area(**LC**), Longchanghe forest area(**LCH**), Qilian forest area(**QL**), Sidalong forest area(**SDL**), Xishui forest area (**XS**), Xiyinghe forest area(**XYH**).

***** Average MAF**(MAF**), Expected allele number(**Ne**), Expected heterozygous number(**He**), Nei_diversity_index(**Ndi**), Number_of_poly_marker(**Npm**), Observed_allele_number(**No**), Observed_heterozygous_number(**Ho**), Polymorphysm_information_content(**PIC**), Shnnon_Wiener_index(**I**).

**Table 3. Genetic diversity index of different populations of Qinghai spruce**

| **Cord**  **Code** | **Accession**  **Name** | **Group** | **Origin** | **Accession**  **Type** |
| --- | --- | --- | --- | --- |
| 1 | DDS97 | DDS（group1） | Dongdashan provenance | Asexual |
| 2 | HX13 | HX（group2） | Haxi | Asexual |
| 3 | HX3 | HX（group2） | Haxi | Asexual |
| 4 | HX7 | HX（group2） | Haxi | Asexual |
| 5 | GC128 | GC（group3） | Gucheng | Asexual |
| 6 | GC14 | GC（group3） | Gucheng | Asexual |
| 7 | GC15 | GC（group3） | Gucheng | Asexual |
| 8 | GC21 | GC（group3） | Gucheng | Asexual |
| 9 | DHS25 | DHS（group4） | Daihuangshan | Asexual |
| 10 | DHS27 | DHS（group4） | Daihuangshan | Asexual |
| 11 | DHS81 | DHS（group4） | Daihuangshan | Asexual |
| 12 | DHK76 | DHK（group5） | Dahekou | Asexual |
| 13 | QL35 | QL（group6） | Qilian | Asexual |
| 14 | LC41 | LC（group7） | Liancheng | Asexual |
| 15 | LC44 | LC（group7） | Liancheng | Asexual |
| 16 | LC45 | LC（group7） | Liancheng | Asexual |
| 17 | LC46 | LC（group7） | Liancheng | Asexual |
| 18 | LC59 | LC（group7） | Liancheng | Asexual |
| 19 | LC63 | LC（group7） | Liancheng | Asexual |
| 20 | LC65 | LC（group7） | Liancheng | Asexual |
| 21 | LCH153 | LCH（group8） | Longchanghe | Asexual |
| 22 | LCH68 | LCH（group8） | Longchanghe | Asexual |
| 23 | XYH70 | XYH（group9） | Xiyinghe | Asexual |
| 24 | XYH71 | XYH（group9） | Xiyinghe | Asexual |
| 25 | XS101 | XS（group10） | Xishui | Asexual |
| 26 | XS110 | XS（group10） | Xishui | Asexual |
| 27 | XS116 | XS（group10） | Xishui | Asexual |
| 28 | XS121 | XS（group10） | Xishui | Asexual |
| 29 | XS135 | XS（group10） | Xishui | Asexual |
| 30 | XS152 | XS（group10） | Xishui | Asexual |
| 31 | XS163 | XS（group10） | Xishui | Asexual |
| 32 | XS88 | XS（group10） | Xishui | Asexual |
| 33 | SDL132 | SDL（group11） | Sidalong | Asexual |

***** Dongdashan forest area(**DDS**), Dahekou forest area(**DHK**), Daihuangshan forest area(**DHS**), Gucheng forest area(**GC**), Haxi forest area(**HX**), Liancheng forest area(**LC**), Longchanghe forest area(**LCH**), Qilian forest area(**QL**), Sidalong forest area(**SDL**), Xishui forest area (**XS**), Xiyinghe forest area(**XYH**).

**Table 4. Assessment table of the genetic diversity of the Qinghai spruce core germplasm**

| Germplasms | No. of accessions | Observed heterozygosity  *（Ho）* | Expected heterozygosity  *（He）* | Nei diversity index  *（H）* | Shanon-Wiener index  *（I）* | polymorphys  m information  content  *（PIC）* |
| --- | --- | --- | --- | --- | --- | --- |
| All | 165 | 0.071-0.616  (0.255) | 0.095-0.500  (0.262) | 0.095-0.502  (0.263) | 0.199-0.693  (0.419) | 0.090-0.375  (0.219) |
| Core | 33 | 0.030-0.900  (0.291) | 0.095-0.500  (0.284) | 0.097-0.509  (0.289) | 0.199-0.693  (0.448) | 0.090-0.375  (0.236) |
| Reserve/% | 20 | 114.12 | 108.40 | 109.89 | 106.92 | 107.76 |

**Table 5.** Distribution of genotypes of the original germplasm and the core germplasm of the Qinghai spruce

| Germplasms | | AA | TT | CC | GG | AG | CT | AC | GT | CG | AT |
| --- | --- | --- | --- | --- | --- | --- | --- | --- | --- | --- | --- |
| All | Distributions | 8506707 | 8514235 | 15260965 | 15216513 | 5424122 | 5447217 | 1939669 | 1928274 | 622781 | 867854 |
|  | Frequencies | 12.49 | 12.51 | 22.41 | 22.35 | 7.97 | 8.00 | 2.85 | 2.83 | 0.91 | 1.27 |
| Core | Distributions | 1509795 | 1511810 | 2685323 | 2674273 | 1150714 | 1158167 | 409782 | 407488 | 130739 | 183883 |
|  | Frequencies | 12.37 | 12.36 | 21.95 | 21.86 | 9.41 | 9.47 | 3.35 | 3.33 | 1.07 | 1.50 |
| Reserve/% |  | 99 | 99 | 98 | 98 | 118 | 118 | 118 | 118 | 118 | 118 |
